# The effectiveness of a men-only supportive expressive group therapy intervention for psychosocial health outcomes in gastrointestinal cancer patients: a 6-month longitudinal study

**DOI:** 10.1186/s12955-021-01687-y

**Published:** 2021-02-05

**Authors:** Devesh Oberoi, Celestina Martopullo, Barry D. Bultz, Linda E. Carlson

**Affiliations:** 1 Cumming School of Medicine, University of Calgary, 3330 Hospital Dr NW, Calgary, AB T2N 4N1 Canada; 2 Cancer Control AB, Tom Baker Cancer Centre Holy Cross Site, 2202 2nd St. SW, Calgary, AB T2S 3C1 Canada

**Keywords:** Supportive expressive group therapy, Gastrointestinal cancers, Men’s SEGT, Psychosical health, Coping, Psychological distress, Quality of life

## Abstract

**Background:**

An increasing number of gastrointestinal cancer (GI) patients suffer from side effects of cancer treatment that can affect their mood states and quality of life. Despite its demonstrated effectiveness in female cancer patients, Supportive Expressive Group Therapy (SEGT) has not been tested in male cancer patients. The current study sought to examine the longitudinal effects of a professionally-led, men-only SEGT on mood states, coping, and quality of life (QoL) in male GI cancer patients.

**Methods:**

A sample of male GI cancer patients (n = 31), at different stages of cancer treatment, was recruited from an ongoing, men-only biweekly GI cancer SEGT. Data were collected at baseline (before or near the beginning of group attendance) and at three months and six months follow-up. All study outcomes were patient-reported and included socio-demographic data as well as validated questionnaires: Profile of Mood States (POMS) for mood states, Functional Assessment of Cancer Therapy-General (FACT-G) for QoL, and Ways of Coping-Cancer Version (WOC) for coping. Linear mixed models were used to examine the change in outcomes over time. Effect sizes were estimated using Cohen’s d.

**Results:**

The Anxiety (*p* = .04; d = 0.70), Depression (*p* = .03; d = 0.93) and Anger (*p* = .04; d = 1.28) subscales of the POMS decreased between baseline and six months. Participants also reported improvements in coping through Distancing (distancing oneself from negative thoughts, being more accepting of the situation, and looking for positives) of the WOC (*p* = .04; d = 0.4) between baseline and six months. There was no change in any of the FACT subscales (QoL) over time.

**Conclusions:**

This is the first study to investigate the effects of a SEGT intervention in male cancer patients. Participation in the intervention was associated with improved mood states and coping in male GI cancer patients; however, there was no change in measures of QoL.

## Background

Gastrointestinal (GI) cancer is a spectrum of diseases and includes cancers of the colon, rectum, pancreas, liver, stomach & esophagus, and accounts for nearly 27% of all cancers worldwide [1]. As a result of biomedical advancements in anticancer treatments, long-term survival is becoming increasingly common in patients diagnosed with GI cancers [2, 3]. However, an increasing number of cancer survivors suffer from late and long-term side effects of cancer treatment that can affect their psychosocial health outcomes [4].

The survivorship challenges faced by GI cancer patients vary and are largely dependent on cancer type, stage of cancer at diagnosis and treatment received. Some of these challenges include bowel dysfunction, eating difficulties, sexual dysfunction, insomnia, urethral infections, and poor mood levels [5]. Furthermore, stoma formation, (an opening on the abdomen that can be connected to the digestive or urinary system to allow waste to be diverted out of the body), increases anxiety around ostomy leakage, spillage, noise, and odour, which may have implications for body image, and social and sexual engagement in many GI cancer patients. When created properly, the stoma can dramatically improve a patient's quality of life; however, complications related to stoma can affect  physical and psychosocial health profoundly [6]. Many of these health issues are considered embarrassing and challenging to discuss with family and friends and may be associated with distress and poor QoL [7]. The risk of future unemployment due to the inability to return to work may further add to the GI cancer patients’ psychological distress [5].

Given the side-effects of treatment, it is not surprising that nearly 1 in 3 patients diagnosed with metastatic GI cancers experience psychological distress and diminished quality of life [8–10]. In some GI cancers such as colon cancer, the incidence of psychological distress is considerably higher, with up to 70% of patients reporting  symptoms of clinical depression [11]. Cancer diagnosis and treatment in these patients can have far-reaching negative consequences, and some patients may even express the need for medical assistance in dying [12]. The lack of social support systems for cancer patients may further contribute to poor psychosocial health outcomes [13]. Psychosocial interventions such as supportive-expressive group therapy (SEGT), were developed to help cancer patients cope with their illness  and improve distress. SEGT helps patients express and manage disease-related emotions, increase social support, enhance relationships with their family and physicians, and improve symptom control [14]. However, most supportive-expressive therapy support groups are offered primarily to women, and studies examining the effects of these interventions  are limited to female patients diagnosed with breast or gynecological cancers [15–18].

In contrast, little attention has been given to studying the structure, function and perceived impact of group formats that address the emotional and existential needs of male cancer patients [19]. One plausible reason is that men are generally reticent to seek help or access health services when experiencing  psychological distress [20, 21]. With seeking help during times of psychological stress being seen as “weak,” by a large porportion of the male population, many choose to suffer in silence[22]. This gap in research makes it challenging to demonstrate what constitutes an effective support intervention for men with cancer.

 Given this, the current study sought to examine the longitudinal impact of an ongoing professionally-led SEGT experience on cancer-related distress, coping and health-related quality of life in male GI cancer patients. Previously, we published qualitative work from interviews with men in this study at baseline, identifying two themes around their motivation for joining the group: affiliation with similar others and learning about coping. Affiliation with other men “in the same boat” was widely anticipated to foster bonding and solidarity through experiential similarity while sharing and comparing cancer experiences was referenced by most men as a way of understanding how others were dealing with cancer [23]. The current study presents longitudinal quantitative data evaluating the patient-reported changes over time in constructs of mood, coping and quality of life. Evaluation of the SEGT through both qualitative and quantitative methods in this group of patients will lay the groundwork for integrating such therapies in the treatment plan of male GI cancer patients[23]. We hypothesized that participation in the SEGT would be associated with an improvement in cancer-related distress, coping behaviour, and QoL, with small to medium effect sizes.

## Methods

### Study design

The current study was a longitudinal study of psychosocial health outcomes (distress, coping and QoL) in male GI cancer patients participating in an ongoing men-only GI cancer SEGT intervention. Data was  collected between May 2015- February 2016. Participants were assessed for their mood states, coping, and health-related quality of life (QoL) at baseline (i.e. before or near the beginning of group attendance (T1) and after three months (T2) and six months (T3) of participation in the group. The aim was to evaluate the patient-reported changes over time in mood, coping and quality of life in these patients.

### Setting

The study was conducted in the Department of Psychosocial Oncology of the Tom Baker Cancer Centre (TBCC) in Calgary, Alberta, Canada. The TBCC is a large tertiary cancer centre that serves the population of Southern Alberta, with a catchment area of approximately 1.6 Million.

### Participants and procedure

#### Eligibility

All men who were attending the ongoing men-only GI cancer SEGT at TBCC.

#### Inclusion criteria

Men who were (1) ≥ 18 years with any GI cancer diagnosis (2) able to communicate in English, (3) attending the ongoing men’s cancer SEGT, were eligible to participate in this study. There were no restrictions on the treatment stage, time since diagnosis, phase of illness, or stage/severity of the disease. Men in the study  could be either on or off treatment.

#### Exclusion criteria

Inability to converse or read/write in English.

#### Recruitment

Study participants were recruited from the ongoing men’s GI cancer SEGT at TBCC that was open to all GI cancer patients of the centre, delivered through the Department of Psychosocial Oncology. Men were referred to this group in the usual manner, which included self-referral, health care team referral, word of mouth and through pamphlets posted in the cancer centre treatment areas. Men potentially eligible for the group were also approached in clinic waiting areas by a Research Assistant, and asked to complete “consent to contact” forms so that a team member could contact them and discuss the group and accompanying optional study. 

New patients who joined the group between May 2015 and February 2016 were eligible to participate in the current study and were recruited through convenience sampling. A total of 219 men were invited to participate by the RA and/or filled out the consent to contact form, of which 213 were eligible and 6 were excluded because they did not have adequate English language skills. Of the eligible men, 177 declined to participate for reasons such as: not interested in the study; did not feel the need for the group; had enough social support; were busy, had pre-scheduled out of town visits; inconvenient time and/or location; poor health; or no specific reasons. Of the 36 remaining patients, five dropped out before the study commenced and 31 completed (response rate = 14.2%) the quantitative assessments at baseline.

Study participation was voluntary and independent of group enrollment, attendance, or entitlement to other psychosocial services at TBCC. Consenting participants were given a package of quantitative assessments to complete at baseline (before starting the group). After attending the group for three months, the participants filled out the same questionnaire package. The same procedures were followed six months after group entry.

### Intervention

The men’s GI cancer SEGT is the only known professionally-led group of its kind, running in Alberta (and beyond) since 2010. The Department of Psychosocial Resources established the SEGT at the TBCC within the GI tumour group in response to an increased number of referrals of distressed GI male cancer patients to the Psychosocial Department, and requests for such a group from male GI patients themselves. Through other formats of psychosocial interventions, GI male cancer patients expressed interest in engaging together in a forum that aims at facilitating information acquisition, provision of emotional support, and development of coping strategies.

The group has been running continually every two weeks for 90 min since its inception, with an average attendance of 12 to 15 men per session. The group adopted a supportive-expressive focus, guided by the principles of SEGT, emphasizing emotional support and shared experiences amongst male GI cancer patients at different stages in their cancer journey. The group is professionally led in dyads by three clinical psycho-oncologists (one female and two male). The group aims to: (1) reduce psychosocial morbidity, (2) facilitate coping/adaptation, and (3) improve quality of life. The structure of the groups was drop-in and ongoing, with the rolling addition of new members and graduation of old members when they no longer wished to attend. Attendance was voluntary and was determined by individual patients as per their needs. The facilitators moderated the discussions and encouraged the participants to talk about their existential concerns.

### Measures

#### Mood states

The Profile of Mood States (POMS) [24] is a 65-item scale that assesses six dimensions of mood: anger, confusion, depression, fatigue, tension, and vigour, as well as total mood disturbance. The scale has a good internal consistency for various dimensions (Cronbach α = 0.84–0.90) as well as good concurrent and discriminant validity [25].

#### Coping

The Ways of Coping Questionnaire- Cancer Version (WOC) [26] is a 53- item questionnaire that assesses coping with regards to five factors: *seeking social support* (e.g. talked to someone to find out more about the situation or to someone who could do something concrete about the problem, getting professional help, accepting sympathy from someone), *focusing on positive* (e.g. grew as a person, found a new faith, prayed, inspired to do something creative), *distancing* (e.g. making light of the situation, went on as if nothing happened, didn’t let it get to them, tried to forget the whole thing, or looked at the silver lining), *cognitive escape-behavioural* (e.g. avoided being with people, came up with different solutions, took a chance and did something risky, criticized or lectured themselves, tried not to act too hastily), and *cognitive escape-avoidance* (e.g. hoped a miracle would happen, took it out on others, wished that situation would go away, use of drugs, smoking, or medication to make themselves feel better, went along with fate). The reliability estimates for these factors have been moderate to high (α ranging from 0.74 to 0.86 for all the factors) [26]. We also conducted a post-hoc analysis to analyze changes over time for each of the individual items of the WOC questionnaire to determine which contributed to the overall change in the subscale over time.

#### Quality of life

Functional Assessment of Cancer Therapy-General [27] is a 27-item scale that assesses four domains of QoL or wellbeing: Physical, Social/family, Emotional, Functional, and overall QoL. The scale has good internal consistency (all Cronbach α ≥ 0.77), test–retest reliability (r = 0.70) as well as construct validity [28].

### Statistical data analysis

#### Data analysis

The sample size was limited to men joining the group over the study period. Participants’ demographic and clinical characteristics were reported using descriptive statistics. To determine the change in the outcomes (mood states, coping and QoL) from baseline to three months and six months of group participation, linear mixed models (LMM) were used. In LMM, time (categorically coded) was included as fixed effect while random intercepts, as well as random slopes, were included as random effects. Unstructured covariance matrix was used. Statistical significance was set at *p* ≤ 0.05. Mixed-level models are appropriate for analysis of this type of data as there is a lack of independence between observations obtained at each time point. In addition, these models are more robust to missing data and unbalanced designs. Chi-square tests were performed to compare the difference in participants who completed the questionnaires at each timepoint with those who completed the baseline but later dropped out at three months and six months. Data were analyzed using SPSS v. 25.

## Results

### Sample characteristics

Thirty-one men completed the questionnaires at baseline, 24 at three months and 13 at six months. The mean age of the participants was 55.96 (± 11.64) years. The majority were Caucasian, and were married or in a common-law relationship. Most participants had completed education beyond high school. Only a few participants were employed. Most participants were diagnosed 6–18 months before the study. Over 50% of the participants had a colorectal cancer diagnosis. Most participants were diagnosed with stage 3 or 4 cancer. Current or prior cancer treatment included chemotherapy, radiation or surgery or a combination of treatments. Characteristics of participants who completed the assessments at T1, T2, and T3 are shown in Table 1.

**Table 1:** Characteristics of participants who completed the assessments at Time1, Time2, and Time3

Variable	T1 n (%); N = 31	T2 n (%) ; N = 24	T3 n (%) ; N = 13
*Age (mean ±SD) 55.96 (±11.64)*			
18–30	1 (3.2)	1 (4.5)	1 (6.7)
31–45	5 (16.1)	5 (22.7)	2 (13.3)
46–60	12 (38.7)	8 (36.4)	6 (40.0)
61–75	13 (41.9)	8 (36.4)	6 (40.0)
*Highest level of education *			
High School	5 (16.1)	3 (13.6)	2 (13.3)
College	14 (45.2)	10 (45.5)	8 (53.3)
University	12 (38.7)	9 (40.9)	5 (33.3)
*Ethnicity*			
Caucasian	31 (93.9)	21 (91.3)	15 (100.0)
African	1 (3.0)	1 (4.3)	0 (0)
Asian	1 (3.0)	1 (4.3)	0 (0)
*Marital Status*			
Married/common law	22 (71.0)	17 (77.3)	12 (80.0)
Single/Divorced/widowed	9 (29.0)	5 (22.7)	3 (20.0)
*Employment Status *			
Employed (Fulltime/part-time)	6 (20.0)	6 (28.6)	4 (28.6)
Unemployed	24 (80.0)	15 (71.4)	10 (71.4)
*Primary GI Tumour type*			
Colorectal	19 (57.6)	14 (60.9)	11 (73.3)
Esophageal	2 (6.1)	1 (4.3)	1 (6.7)
Stomach	1 (3.0)	1 (4.3)	1 (6.7)
Pancreatic	6 (18.2)	3 (13.0)	1 (6.7)
Liver	1 (3.0)	1 (4.3)	0(0)
Bile duct	1 (3.0)	1 (4.3)	0(0)
*Tumour stage*			
1	2 (8.3)	2 (10.0)	2 (15.4)
2	3 (12.5)	3 (15.0)	2 (15.4)
3	6 (25.0)	5 (25.0)	5 (38.5)
4	13 (54.2)	10 (50.0)	4 (30.8)
*Time since diagnosis *			
≤6 months	13 (44.8)	8 (38.1)	5 (35.7)
6.1–12 months	7 (24.1)	6 (28.6)	3 (21.4)
12.1–18 months	3(10.3)	3 (14.3)	2 (14.3)
18.1–24 months	3(10.3)	1 (4.80	1 (7.1)
>24 months	3(10.3)	3 (14.3)	5 (21.4)
*Multiple cancer diagnoses*			
No	23 (74.2)	17 (77.3)	11 (73.3)
Yes	8 (25.8)	5 (22.7)	4 (26.7)
*Current treatment *			
Surgery	5 (16.1)	4 (18.2)	3 (20.0)
Chemotherapy	19 (61.3)	12 (54.5)	7 (46.7)
Radiation therapy	1 (3.2)	1 (4.5)	1 (6.7)
Upcoming treatment	1 (3.2)	1 (4.5)	–
*Past treatment*			
Surgery	16 (48.5)	11 (50.0)	9 (60.0)
Chemotherapy	10 (32.3)	9 (40.9)	8 (53.3)
Radiation therapy	6 (19.4)	5 (22.7)	5 (33.3)

*T1 = baseline, T2 = 3 months and T3 = 6 months

N, total number of participants

The maximum number of group sessions attended by the participants over the study period was 13, and the mean number attended was 5.56 (± 3.68).

The reasons for dropouts were reported as due to the following:

- **At 3 months:** (n = 8 drop outs)

Deceased (n = 2); lack of time or not needing group support at that time (n = 2); viewing group as a downer (n = 1); did not consider himself suitable for the group (n = 1); looking for a group with only a palliative membership (n = 1); poor health (n = 1).

- **At 6 months:** (n = 11 drop outs)

Deceased (n = 5); moved out of country/province (n = 3) deteriorating physical health (n = 2); returned to work (n = 1).

The results of chi-square tests showed no differences in participants who completed the follow-up questionnaires at T2 (n = 24) and those who dropped out with regards to age (*p* = 0.37), education (*p* = 0.82), ethnicity (*p* = 0.38), marital status (*p* = 0.23), employment status (*p* = 0.07), tumour type (*p* = 0.81), tumour stage (*p* = 0.71), time since diagnosis (*p* = 0.20), or multiple cancer diagnoses (*p* = 0.54). Likewise, there were no differences in participant characteristics between those who completed the follow-up questionnaires at T3 (n = 13) and those who dropped out with regards to age (*p* = 0.74), education (*p* = 0.67), ethnicity (*p* = 0.37), marital status (*p* = 0.28), employment status (*p* = 0.27), tumour type (*p* = 0.81), tumour stage (*p* = 0.74) time since diagnosis (*p* = 0.35), or multiple cancer diagnoses (*p* = 0.92).

### Change in outcome scores

Participants’ mean scores for POMS, WOC, and FACT-G at T1, T2 and T3 are shown in Table 2.

**Table 2:** Participants’ mean subscale scores at Time1, Time2, and Time3

	Range	Time 1			Time 2			Time 3		
		M	SD	n	M	SD	n	M	SD	n
*POMS-65*										
Tension-anxiety	0–36	9.48	7.35	31	8.50	7.48	24	5.92	6.10	13
Depression-dejection	0–60	10.45	10.46	31	8.46	10.27	24	5.15	6.05	13
Anger-hostility	0–48	8.97	7.74	31	7.83	8.62	24	6.15	8.43	13
Vigor	0–32	15.45	6.89	31	17.46	6.72	24	15.92	7.56	13
Fatigue	0–28	8.61	5.51	31	8.63	6.71	24	10.00	7.45	13
Confusion	0–28	8.39	5.96	31	1.94	3.15	24	7.920	5.34	13
Total mood disturbance	0–200	30.49	39.32	31	22.78	37.9	24	19.20	32.69	13
*WOC-CV*										
Seek & use social support	0–32	22.03	9.11	29	24.57	7.49	23	20.17	6.10	12
Cognitive escape avoidance	0–16	14.72	6.54	29	14.54	5.96	24	13.67	5.01	12
Distancing	0–24	23.34	7.30	29	27.17	6.60	23	26.17	5.49	12
Focus on positive	0–24	12.55	7.38	29	15.65	6.74	23	13.25	5.80	12
Behavioural escape avoidance	0–12	10.34	4.80	29	11.13	4.43	23	10.25	4.83	12
*FACT-G*										
Physical wellbeing	0–28	19.51	5.44	31	21.38	4.24	24	19.23	5.63	13
Social wellbeing	0–28	20.48	6.12	31	20.71	6.24	24	20.41	4.91	13
Emotional wellbeing	0–28	16.77	5.50	31	18.67	3.89	24	19.62	3.07	13
Functional wellbeing	0–24	16.57	6.30	31	18.63	5.29	24	20.23	5.26	13
Total score	0–108	73.35	18.42	31	79.38	12.55	24	79.49	14.31	13

FACT-G, Functional assessment of cancer therapy -general; POMS-65, Profile of mood states; WOC, Ways of coping questionnaire

*T1 = Baseline; T2 = 3 months and T3 = 6 months

#### Mood states

**Fig. 1:**
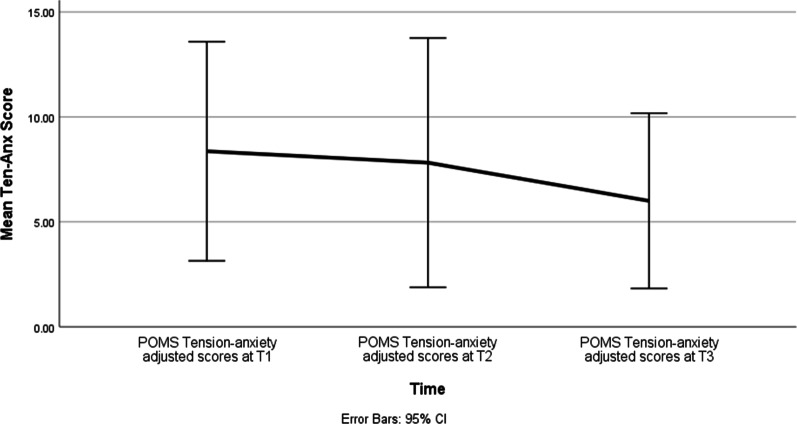
Mean POMS Tension-anxiety scores at baseline (T1), three months (T2), and six months (T3)

**Fig. 2:**
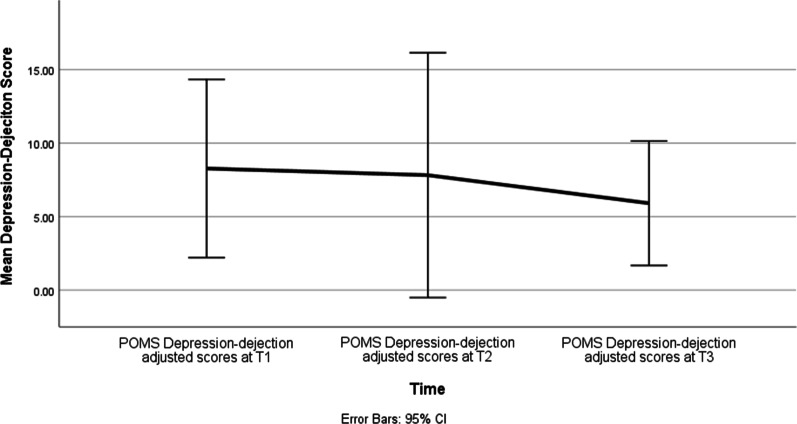
Mean POMS Depression-dejection scores at baseline (T1), three months (T2), and six months (T3)

**Fig. 3:**
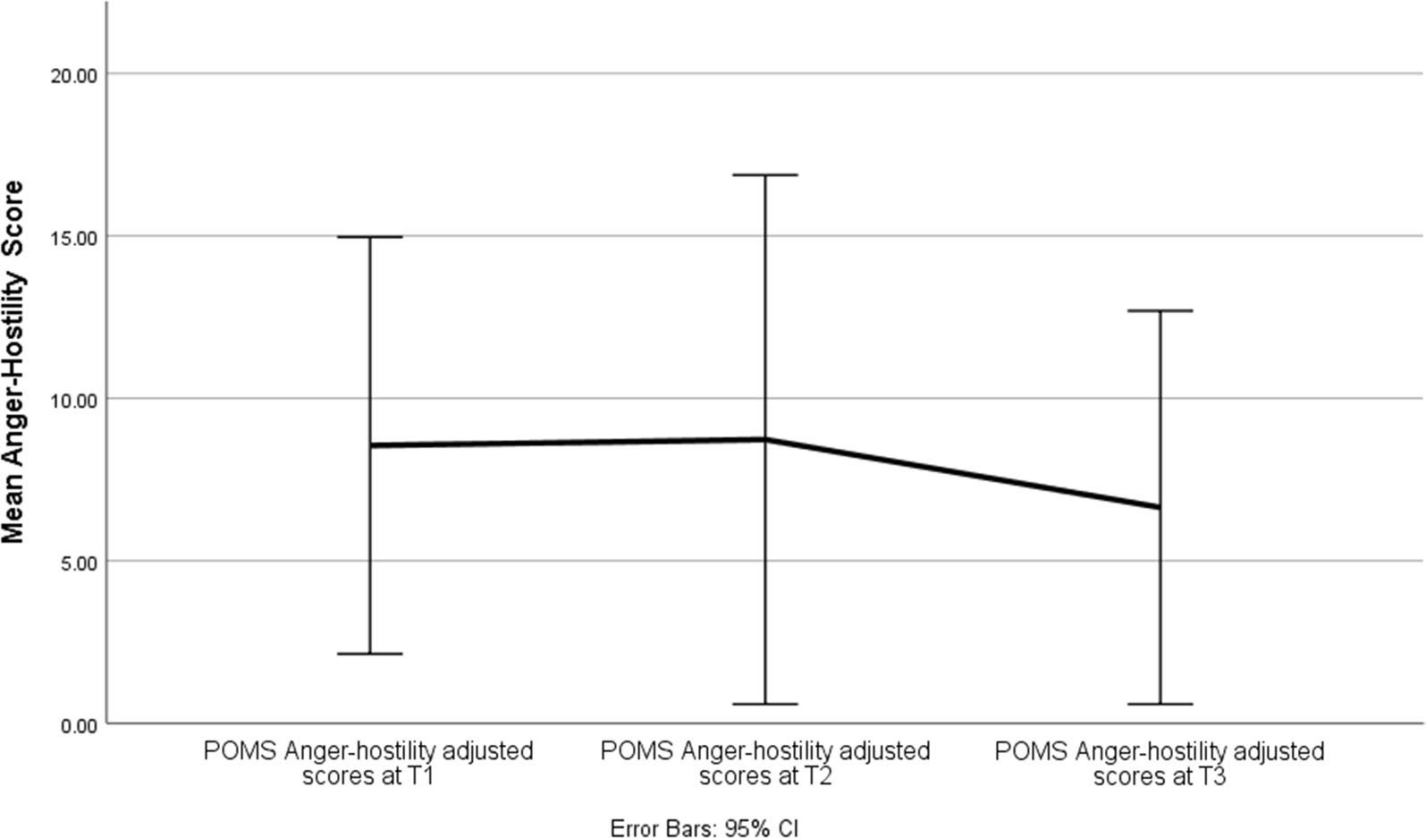
Mean Anger-Hostility scores at baseline (T1), three months (T2), and six months (T3)

Effect of time was significant for Anxiety (F = 4.34, *p* = 0.025), Depression (F = 3.82, *p* = 0.037) and Anger (F = 3.97, *p* = 0.034) subscales. The Anxiety subscale scores improved over time between T1 and T3 (*p* = 0.04; d = 0.70) as well as between T2 and T3 (*p* = 0.01; d = 0.90) (Fig. 1). The Depression subscale scores improved over time between T1 and T3 (*p* = 0.03; d = 0.93) (Fig. 2). The Anger subscale scores improved over time between T1 and T3 (*p* = 0.04; d = 1.28), as well as between T2 and T3 (*p* = 0.03; d = 0.7) (Fig. 3). There was no effect of time on any of the other POMS subscales or the overall total mood disorder (TMD) (Table 3).

**Table 3:** Change in mood states over time from Time1 to Time3

Outcome (POMS)	Parameter	Estimate	SE	df	t	Sig.	95% CI
Tension-Anxiety	Intercept	7.04	1.05	30.72	6.72	1.67E-07	4.90–9.17
	T1	2.24	1.04	25.02	2.15	0.04	.10–4.37
	T2	1.96	0.7	21.04	2.81	**0.01**	.51–3.41
	T3	0	0	–	–	–	–
Depression	Intercept	6.5	1.14	25.05	5.68	6.37E−06	4.15–8.86
	T1	3.53	1.42	14.79	2.48	**0.03**	49–6.57
	T2	2.55	1.35	23.1	1.89	0.07	− 5.58
	T3	0	0	–	–	–	–
Anger	Intercept	6.45	1.25	33.41	5.16	1.11E−05	3.91–8.99
	T1	2.31	1.02	20.9	2.25	**0.04**	.18–4.43
	T2	1.82	0.76	20.69	2.4	**0.03**	.24–3.40
	T3	0	0	–	–	–	–
Vigour	Intercept	15.78	1.7	18.52	9.27	2.22E−08	12.21–19.35
	T1	− 0.29	1.74	21.31	− 0.16	0.87	− 7.22
	T2	1.3	1.47	14.4	0.89	0.39	− 6.28
	T3	0	0	–	–	–	–
Fatigue	Intercept	10.31	1.77	16.14	5.82	2.52E−05	6.56–14.06
	T1	− 1.77	1.75	16.16	− 1.02	0.32	− 7.39
	T2	− 1.54	− 1.54	15.41	− 0.89	0.39	− 7.39
	T3	0	0	–	–	–	–
Confusion	Intercept	7.83	0.86	31.17	9.08	2.87E−10	6.07–9.58
	T1	0.42	0.87	24.4	0.49	0.63	− 3.58
	T2	− 0.84	0.53	17.22	− 1.57	0.13	− 2.25
	T3	0	0	–	–	–	–
POMS-TMD	Intercept	22.39	5.9	26.13	3.8	0	10.27–34.51
	T1	6.85	6.31	21.15	1.09	0.29	− 26.25
	T2	2.63	5	19.68	0.53	0.6	− 20.87
	T3	0	0	–	–	–	–

*T1 = baseline, T2 = 3 months and T3 = 6 months

POMS-TM, Profile of mood states-total mood disorder

#### Coping

**Fig. 4:**
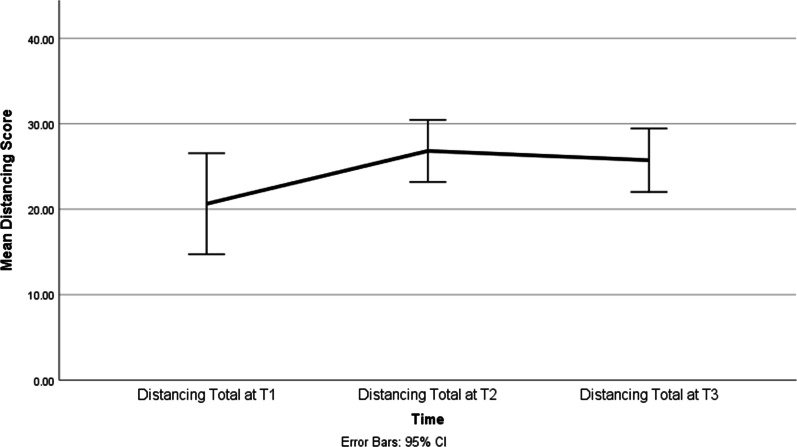
Mean WOC-Distancing score at baseline (T1), three months (T2), and six months (T3)

There was no effect of time on seeking social support, focusing on the positive, cognitive escape-behavioural, and cognitive escape-avoidance coping; however, the effect of time on Distancing was significant (F = 6.69, *p* = 0.007). The scores on the distancing subscale increased over time between T1 and T3 (*p* = 0.04; d = 0.4) (Fig. 4) but not between T2 and T3 (Table 4**).** Interestingly, in the *Distancing* subscale only three items changed over time (*Didn't let it get to me; Treated the illness as a challenge; Tried to keep my feelings from interfering)*. We also found significant changes over time in the following items: *Let my feelings out somehow; Tried to find out as much as I could* (Seek and Use Social Support subscale); *Prayed* (Cognitive Escape Avoidance subscale); *Thought of how a person I admire would act* (Focusing on Positive subscale) (Table 5).

**Table 4:** Change in coping over time from Time1 to Time3

Outcome	Parameter	Estimate	SE	df	t	Sig	95% CI
Behavioral escape avoidance	Intercept	10.71	1.01	20.47	10.63	8.70E−10	8.61–12.81
	T1	− 0.56	0.95	18.82	− 0.59	0.56	− 2.55 1.42
	T2	0.44	0.84	14.34	0.52	0.61	− 1.36 2.24
	T3	0	0	–	–	–	–
Cognitive escape avoidance	Intercept	15.17	1.03	21.72	14.71	8.96E−13	13.03–17.31
	T1	− 0.56	1.3	22.88	− 0.43	0.67	− 5.39
	T2	− 0.13	0.75	14.78	− 0.18	0.86	− 3.22
	T3	0	0	.	.	.	.
Distancing	Intercept	26.95	1.36	13.61	19.75	2.07E− 11	24.02–29.89
	T1	− 3.42	1.56	19.45	− 2.19	**0.04**	− 6.84
	T2	0.82	1.58	11.57	0.52	0.62	− 6.92
	T3	0	0	–	–	–	–
Focus on positive	Intercept	14.24	1.38	18.75	10.29	0	11.34–17.14
	T1	− 1.53	1.22	18.57	− 1.25	0.23	− 5.11
	T2	0.94	0.97	12.74	0.97	0.35	− 4.19
	T3	0	0	–	–	–	–
Seek and use social support	Intercept	21.63	1.4	18.28	15.4	6.54E−12	18.68−24.58
	T1	0.62	1.41	17.36	0.44	0.67	− -5.93
	T2	2.8	1.35	15.16	2.08	0.06	− 5.75
	T3	0	0	–	–	–	–

*T1 = baseline, T2 = 3 months and T3 = 6 months

**Table 5:** Items in Ways of Coping (WOC-CV) questionnaire that underwent significant changes over time from Time1 to Time3

Item #	Subscale	Item	Changes in time points (Sig. change at *p* < 0.05)
20	Seek and use social support	Let my feelings out somehow	T1 < T2 (*p* = 0.03); and T2 < T3 (*p* = 0.04)
49	Seek and use social support	Tried to find out as much as I could	T2 < T3 (*p* = 0.02)
30	Distancing	Didn't let it get to me; refused to think about it	T1 < T2 (*p* = 0.01); and T1 < T3 (*p* = 0.04)
40	Distancing	Tried to keep my feelings from interfering	T1 < T3 (*p* = 0.02)
50	Distancing	Treated the illness as a challenge	T1 < T2 (*p* = 0.03); and T2 < T3 (*p* = 0.04)
44	Cognitive Escape Avoidance	Prayed	T1 < T3 (*p* = 0.04)
47	Focusing on positive	Thought of how a person I admire would act	T1 < T3 (*p* = 0.02)

*T1 = baseline; T2 = 3 months and T3 = 6 months

#### QoL

The QoL scores for social wellbeing (SWB), emotional wellbeing (EWB), functional wellbeing (FWB) and the total FACT-G of our sample at three months and six months were statistically similar (*p* > 0.05) to baseline scores. However, there was a significant effect of time on physical well-being (PWB) (F = 4.26; *p* = 0.03), which deteriorated between T2 and T3. There was no significant difference between PWB at T1 and T3, but there was a statistically significant difference between the PWB at T2(M = 21.38 [4.41] and T3 (m = 19.13 [5.63] (Table 6).

**Table 6:** Change in quality of life (QoL) over time from Time1 to Time3

Outcome (FACT)	Parameter	Estimate	SE	df	t	Sig	95% CI
Physical well being	Intercept	18.67	1.31	17.33	14.17	0	15.89–21.44
	T1	0.92	1.49	21.19	0.62	0.54	− 6.2
	T2	2.7	1.07	12.11	2.52	0.03	.37–5.03
	T3	0	0				
Emotional well being	Intercept	18.55	0.75	15.85	24.67	4.49E−14	16.96–20.15
	T1	− 1.56	0.98	24.04	− 1.58	0.13	− 4.07
	T2	− 0.43	0.78	16.12	− 0.55	0.59	− 3.3
	T3	0	0				
Sociall well being	Intercept	20.16	0.95	28	21.12	9.71E−19	18.20–22.11
	T1	0.48	0.59	14.87	0.81	0.43	− 2.51
	T2	0.54	1.19	17.46	0.46	0.65	− 4.99
	T3	0	0				
Functional well being	Intercept	18.38	1.3	15.56	14.16	2.65E−10	15.63–21.14
	T1	− 1.62	1.33	17.01	− 1.21	0.24	− 5.63
	T2	− 0.16	1.37	18.41	− 0.11	0.91	− 5.75
	T3	0	0	–	–	–	–
FACT* Total	Intercept	74.83	3.22	17.89	23.24	8.19E−15	68.07–81.61
	T1	− 0.77	3.3	19.03	− 0.23	0.82	− 13.82
	T2	3.5	3.14	15.84	1.11	0.28	− 13.32
	T3	0	0	–	–	–	–

FACT* Functional assessment of cancer therapy

*T1, baseline, T2, 3 months and T3, 6 months

## Discussion

The current study is the first to report outcome data on the association  of SEGT with psychosocial health outcomes in male GI cancer patients who participated in an ongoing professionally-led men-only cancer SEGT group. The program was based on a drop-in format and the mean number of classes attended by the participants was 5.56 (± 3.68).We found that participants’ mood states improved over time with regard to feelings of *anxiety, depression*, and *anger*. The effect size of the change in anxiety scores was medium (d = 0.7) between baseline and six months, and was large (d = 0.9) between three and six months, suggesting that participation for a longer duration for those who continued to attend the group resulted in more significant anxiety reduction. Likewise, the effect size was large for change in depression scores (d = 0.9) as well as anger scores (d = 1.28) between baseline and six months and was medium (d = 0.7) for change in anger scores between three and six months of group participation. Participants also experienced an improvement in *distancing,* a sign of potential growth in coping over time, discussed in more detail below. The change in the *distancing* subscale of coping between baseline and six months had a small (d = 0.4) effect size. These patterns suggest an immediate and lasting effect on feelings of depression and anger upon joining the group, with enhanced effects on managing anxiety over time. We did not observe any improvement in QoL, measured with the FACT scale. Overall, our findings are consistent with past research focusing on the role of SEGT in reducing distress and disturbance in mood states in female cancer patients, especially anger and symptoms of cancer-related stress [18, 29].

Cancer patients must also cope with the stresses induced by chronic health impairment and disability, fatigue, and pain resulting from cancer treatment [30]. These effects further contribute to emotional distress. Anger is particularly common in those struggling with the initial stage of shock and denial, and the uncertainty of their cancer prognosis; anger is also more commonly expressed by men than women, while women are more apt to endorse feelings of sadness or depression [31]. A men-only forum provides a secure medium for participants to share cancer-related experiences with less fear of embarrassment than mixed-sex groups [32]. It has been argued that men often become more expressive when there is a perceived degree of autonomy, and their feelings of internal control are not threatened [33]. In our previous study with the same set of men, participants had cited the men-only composition as one of the primary reasons for joining the group in our previous qualitative study [23].

Despite strong similarities with literature on the effects of SEGT in improving psychosocial health outcomes in female cancer patients, we also found some differences between our findings and other studies. Papastergiou et al. [18] reported improvement in anger in mostly female cancer patients following participation in a SEGT program, but no significant improvement in depression and anxiety. Another study on the use of SEGT in Chinese breast cancer patients reported the intervention was ineffective in improving symptoms of anxiety and depression, which the authors speculate may have been due to low initial distress levels in women participating in the trial [34]. A plausible explanation for many of these differences could be the gender effects on externalization of distress and patients’ emotional and behavioral responses to these symptoms [31]. Evidence suggests that men struggling to adhere to the norms of hegemonic masculinity are more likely to express their emotional and psychological distress in the form of externalizing symptoms such as anger, irritability, and self-distraction [21, 35]. It is one of the ways men typically respond to distressing situations that show a distinct gender difference in the embodiment of depression. In essence, women tend to get sad while men get angry [21, 31]. In their analysis of the National Comorbidity Survey Replication using the Male Symptom Scale (MSS), Martin et al. [31], found that men endorsed anger as a symptom of depression at significantly higher rates than women. Additionally, men had a higher prevalence of depression compared to women in their study. Women, on the other hand, endorsed symptoms such as stress, irritability, sleep problems, and loss of interest in things they usually enjoyed at significantly higher rates than men [31]. We hypothesize that reduction in anger may mediate improvement in distress levels in men, whereas addressing distress levels in women may require interventions that focus more on stress, sleep and irritability. Given the strong relationship between anger and depression in men[36], the SEGT intervention, which has been shown to reduce the feelings of anger over time, could be particularly impactful in alleviating the symptoms of depression in men. However, this needs to be investigated in future studies.

There was a significant improvement in the *distancing* subscale of the Coping measure in our study; however, none of the other subscales of the Coping measure changed over time. Because the distancing subscale includes items around avoidance of difficult emotions as well as items implying further engagement with emotions, we conducted a post-hoc analysis to analyze changes over time for each of the 12 items of the subscale to determine which contributed to the overall change in the subscale over time. Interestingly, only three items changed over time (*Didn't let it get to me; Treated the illness as a challenge; Tried to keep my feelings from interfering)*. These items seem consistent with masculine coping skills and are supportive of the constructs of hegemonic masculinity, which perpetuate men’s image as strong, resilient, and invulnerable and, at times, discourages health-positive behaviours or seeking help among men. Further, in our post-hoc analysis, we analysed the change over time for all items of the WOC measure, to determine if other items changed over time even though the subscale scores did not. We found significant changes over time in the following items: *Let my feelings out somehow; Tried to find out as much as I could* (Seek and Use Social Support subscale); *Prayed* (Cognitive Escape Avoidance subscale); *Thought of how a person I admire would act* (Focusing on Positive subscale). Change over time in these items suggests that SEGT facilitated coping by encouraging men to open-up about their feelings within the men’s group, which is in line with the *Expressive* component of the group. As well they seem to have been using the other group participants as a source of support, information and positive role models, also in accordance with SEGT principles. This post-hoc exploration of the coping scale helps to better unpack the benefits specific to men in this therapeutic format, providing ideas and direction for future research.

Although most cancer patients often use more than one coping method, *Distancing* has been reported to be the most common [26]. Older age may reduce perceptions of cancer as a threat and the understanding of the number of coping options one has, with many patients adopting *Distancing *as their primary coping method [26]. This is consistent with our study as around 42% of our participants were over the age of 60 years. Overall, our findings are compatible with the goals of SEGT, which are to enhance the individual’s ability to cope with the disease, facilitate self-assessment, bonding with others and overcoming feelings of loneliness among patients [37]. Enhancement of coping skills can help patients reduce their sense of helplessness by developing a more active repertoire of responses to stressors [38]. Improvement in the coping behaviour in our study also concurs with participants’ primary reason to join the group, which was primarily to learn to cope better.

We did not observe an association between participation in the intervention and any of the FACT subscales, which includes physical, emotional, social and functional QoL and overall wellbeing. Our findings were echoed by other studies that observed no significant effects on QoL in female cancer patients participating in SEGT [39]. One likely explanation is that most of our participants were late-stage cancer patients, and some were still undergoing cancer treatment, presumably resulting in diminishing QoL and little possibility for improvement. Patients with advanced and terminal cancer suffer from various physical symptoms and often experience a continuous decline in physical and cognitive functions. The side-effects of cancer treatment can further reduce pateints’ QoL. Our participants were mostly stage-3 and 4 cancer patients who were likely experiencing deteriorating health, and no downward changes in other measures may be interpreted as a good sign for the effects of the intervention. These findings should also be viewed in the context of participants’ reasons to join the SEGT, which were primarily affiliation with similar others and learning new ways of coping from other men in the group [23], instead of expecting any improvement in their QoL through group participation. Since this measure may not align with participants’ reasons to join the group, it makes sense that we did not observe any substantial change in these outcomes. The findings suggest that for any noticeable change in the results, the clinical outcomes under investigation should be consistent with participants’ motivation for joining the group.

Despite significant findings reported in this study, there are some noteworthy limitations. First, this was a small study and was potentially underpowered to estimate the treatment effect for all the outcomes under investigation. Second, the participants were required to complete a battery of measures at multiple assessment points. This assessment burden may have precluded recruitment of participants who believed they were unable to meet these requirements, as well as contributed to drop out at follow up assessments. Third, this was a pre-post study, and we did not have a control/comparison group and, therefore, no assessment of the natural course of changes in these outcomes in the absence of such intervention was possible. Thus, it is difficult to determine whether observed changes were due to attending the group itself, or other outside factors, which could include the natural progression of symptoms and disease status, changes in treatment regimens, and the effects of repeated testing. Furthermore, we did not evaluate the dose–effect relationship between the number of sessions attended and the changes in health outcomes. The knowledge of an effective dose of the intervention with regards to patients adherence and burden is critical for the success of the intervention [40]. The improved understanding of dose–response relationships with regards to frequency, amount and duration of sessions in future studies could help to design more efficient SEGT interventions for greater effects in health outcomes [40].

Lastly, only 14.2% of eligible patients invited participated in the study. It is possible that only men who were self-motivated to participate in the intervention made up the majority of the sample, which may impact on the external validity of the findings. We also did not interview men who declined to participate in the study. The reasons for low uptake need to be investigated to inform the design and development of men’s SEGT interventions in future.

## Conclusions

Our preliminary findings in a men’s GI SEGT show that participation in supportive-expressive group therapy is associated with improvement in distress and coping behaviour in male GI cancer patients. Results also suggest that SEGT may work differently in men compared to women due to the externalization of depression as anger. Future randomized studies with larger samples and in other cancer types are needed to confirm the validity of the findings. The misalignment between men’s reasons to join the group and in some of the measured health outcomes (QoL) yield valuable input for future research. Findings also suggest that undertaking formative qualitative research before doing randomized effectiveness studies might help inform the researchers about suitable health outcomes to be measured.

## Data Availability

The datasets used and/or analysed during the current study are available from the corresponding author on reasonable request.
